# A Performant Web-Based Visualization, Assessment, and Collaboration Tool for Multidimensional Biosignals

**DOI:** 10.3389/fninf.2019.00065

**Published:** 2019-09-24

**Authors:** Maximilian Beier, Thomas Penzel, Dagmar Krefting

**Affiliations:** ^1^Center of Sleep Medicine, Charité–Universitätsmedizin Berlin, Berlin, Germany; ^2^Center for Biomedical Image and Information Processing, University of Applied Sciences, Berlin, Germany; ^3^Department of Medical Informatics, University Medical Center Goettingen, Göttingen, Germany

**Keywords:** biosignals, visualization, collaboration, EDF, PSG

## Abstract

Biosignal-based research is often multidisciplinary and benefits greatly from multi-site collaboration. This requires appropriate tooling that supports collaboration, is easy to use, and is accessible. However, current software tools do not provide the necessary functionality, usability, and ubiquitous availability. The latter is particularly crucial in environments, such as hospitals, which often restrict users' permissions to install software. This paper introduces a new web-based application for interactive biosignal visualization and assessment. A focus has been placed on performance to allow for handling files of any size. The proposed solution can load local and remote files. It parses data locally on the client, and harmonizes channel labels. The data can then be scored, annotated, pseudonymized and uploaded to a clinical data management system for further analysis. The data and all actions can be interactively shared with a second party. This lowers the barrier to quickly visually examine data, collaborate and make informed decisions.

## 1. Introduction

Biosignals are an integral part of different medical fields like neurology, cardiology, pneumology or sleep medicine (Semmlow, 2012). These disciplines strongly rely on the visual interpretation of biosignals to find pathological patterns and obtain information about a patient's condition (Naït-Ali, 2009). The signals are acquired digitally and assessed by researchers or clinicians via a variety of software solutions. Since the causes of diseases can be manifold, diagnosis increasingly require interdisciplinary knowledge and cooperation (Körner, 2010). In general, however, non-commercial solutions only allow a single user. Thus, if one wants to consult another person, he or she has to be physically available to work in front of the same monitor and finger point to interesting aspects of the biosignals. And although data can be uploaded to private or public online repositories (Goldberger et al., 2000; Krefting et al., 2013) or viewed using screen-sharing methods or remote desktop tools (that are barely found in hospitals due to security reasons), this highly interactive kind of discussion on biosignals has currently no virtual counterpart. Furthermore, since hospitals work with sensitive data, their computer systems are restrictive to maintain integrity and users usually cannot install their own software (Anderson, 1996). They are thereby bound to certain computers and the programs available on them, which further hinders collaborative work between disciplines. For the presented application, sleep medicine was chosen as the primary application area, as it covers a wide range of biosignals and is inherently interdisciplinary.

### 1.1. Sleep Medicine

The comprehensive analytical method to investigate the course, depth and quality of sleep is the so-called polysomnography (PSG) (Ibáñez et al., 2018). They usually span a whole night sleep of about 8 h and contain multiple biosignals like brain activity (EEG), eye movement (EOG), muscle tone (EMG), and cardiac activity (ECG). The AASM Manual for the Scoring of Sleep (Berry et al., 2017) recommends a minimum of 16 signals for a routine PSG^1^, but recordings can contain up to 40 different sensors (Roebuck et al., 2014). A typical PSG today encompasses about 300 MB of biosignal data. Sleep is a dynamic process and can be divided into awake (W), light (N1, N2), deep (N3), and REM (R) sleep (Iber et al., 2007). These are traversed in typical sleep patterns over the night and are assigned by reviewing the recorded signals in 30 s windows, so-called epochs. This results in a graph called hypnogramm. In addition, special events can be marked, e.g., cessation of breathing (apnea) or limb twitching. Based on this data, a diagnosis can be made and the referral to a specialist recommended.

## 2. Requirements and Concept

The aim of our work is a solution that enables researchers and clinicians to inspect and evaluate biosignals in a familiar user interface and easily consult another person in order to work together, interactively receive feedback and jointly gain insights. In qualitative interviews with researchers and technical staff from different sleep labs in Germany, we have deduced the following requirements. The solution should:

- Work location independent
  The application should run on common desktop computers and mobile devices without installation.
- Be performant
  The user interface should provide a fast initial rendering and allow for uninterrupted scrolling through the data. Its performance should be independent of a file's size.
- Support assessment
  The user interface should mimic established applications and allow to assess a PSG, in particular to create a hypnogramm and mark events. Events need to be imported and exported.
- Allow for collaboration
  Multi-user operation and live interactive data exchange with at least one other authenticated party must be possible without additional tools or browser plugins.
- Protect patient data
  Identifying information should only be transmitted in pseudonymized form by default.
- Support most browsers
  All modern browsers like Chrome, Firefox, Safari and Edge must be supported. IE11 should be supported as much as possible.
- Support most biosignal devices
  The application should support a file format that most devices are able to export.
- Enable advanced analysis
  It should be possible to employ external analysis tools, such as automatic preprocessing of the data.

To fulfill requirement (a), a web-based application is the method of choice. All modern desktop computers and mobile devices ship with a pre-installed web browser, that is capable of executing arbitrary JavaScript (JS) code. An implementation as a Single Page Application in HTML, CSS, and JS ensures that it works on any of these systems without local installation. Interactive assessment (c) can also be realized with these technologies. Requirement (b) is addressed by testing different data loading methods and rendering approaches. For collaboration (d) over the web, WebRTC is used. Pseudonymization (e) can be achieved by replacing identifying data in the transfer stream with locally generated substitutes. The solution should also work offline if used alone to guarantee that there is no data-leakage. To fulfill (f), only methods currently supported by all modern browsers are implemented. Most software tools that come with biosignal devices allow to export data to the European Data Format^2^ (EDF). Therefore, the solution is based on EDF (g). Data transfer to XNAT, a popular biomedical research platform, and ingestion of analysis results from XNAT is supported (h).

## 3. Related Work

Most commercial PSG devices store recordings in proprietary formats that can be visualized with also proprietary software provided by the manufacturers, e.g., Domino (Somnomedics), Noxturnal (ResMed), or Sleepware G3 (Philips). They all support assessment (c), are performant (b) and can import and export EDF (g) but need to be installed (a) and don't offer collaboration functionality (d).

With regard to biosignal viewers there are several open-source tools to visualize EDF files listed on the EDF website. Among them Sleep (Combrisson et al., 2017) and SigViewer for Windows, macOS and Linux, or Polyman for Windows (Kemp and Roessen, 2007).

Furthermore, an increasing number of online EDF viewers are available^3^, including commercial services for sleep scoring training like the AASM Sleep ISR. However, they are all interfaces to online data repositories, require special server-side applications to parse EDF into a custom format, or cannot be used for own data.

In 2015 our proof-of-concept version of a purely web-browser-based EDF parser and visualizer was published as part of a biosignal research infrastructure (Beier et al., 2017). It supports requirements (a) and (e)–(g), but is relatively slow and offers no further features. In 2017, Bilal Zonjy released a web browser-based EDF viewer^4^ that also supports local and remote EDF files, supporting requirements (a), (f), (g), and (h). Justus Schwabedal released a similar application^5^ in 2018 that additionally offers automatic sleep stage scoring (c). None of those solutions allows for remote collaboration. On the other hand, several solutions for web-based collaboration have been proposed, like CBRAIN (Sherif et al., 2014) for neuroimaging research, P2Care (Maglogiannis et al., 2006; Andriopoulou et al., 2015) for general real-time teleconsultation, or BUCOMAX (Puel et al., 2014) and HERMES (Andrikos et al., 2019) for radiologists. But these solutions do not support biosignal recordings.

## 4. Methods

As stated in requirement (b), the application should show at least the performance as existing solutions for the user. This includes two aspects: At application startup, the First Meaningful Paint (FMP), i.e., the time from the start of the application to the first rendered graphs, is considered the respective performance measure. We assume FMP ≤ 2 s as acceptable. The second measure is the time the application needs to update these graphs due to user input, for example while scrolling on the time axis; the latency L. This does not only include the applications work but also the browsers work like layouting, painting, and handling user input. If the application code uses too much time, the rendering process is blocked, frames are dropped and the response to user interaction is delayed, which leads to visible inconsistencies. We assume a L ≤ 100 ms response time as good, as this is typically an accepted time delay in computer networks. As the application is for inspection of static data and is typically not in a time critical context we assume L ≤ 200 ms as acceptable. A lower limit for L would be the frame rate of the monitor (60 Hz), resulting in *L*_*min* = 16 ms. Both RAM consumption as well as CPU load can contribute to low performance, and are both considered.

### 4.1. Data Formats

EDF (Kemp et al., 1992) [and its successor EDF+ Kemp and Olivan, 2003] is a free and open format designed to store time series of multiple signals and is the de facto standard for biosignal recordings. It consists of three parts: a static header, a dynamic header, and the signal data.

The static header contains metadata about the recording, the recorded channels, and the patient. Only the “local patient identification” field is intended to contain identifying information like name and birth date.

The dynamic header includes metadata for each channel. EDF specifies the structure of the metadata and the signals, but not the signal description labels. EDF+ added obligatory standard names and naming schemes (Kemp et al., 2003), but they are not enforced. Due to low compliance, several variations for the same signal can be found in different devices. They might differ slightly (e.g., “C4-A1” = “EEGC4-A1” = “EEG C4-A1”) or strongly (e.g., “LEGBEINLI” = “EMG LAT”). Most of the labels are acquired by the staff in the clinical routine with a limited set of devices in a lab. However, they are problematic in automated environments or when working in multicenter studies. Therefore, a list of commonly used variants of channel names and their equivalent in the standard has been manually compiled (Beier et al., 2017). It currently lists 350 variations of 88 standard signals, among them 47 EEG channels, 11 EMG channels and 9 EOG locations).

**Figure 1:**
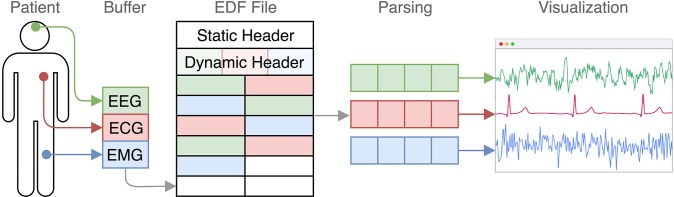
EDF in the context from measurement to visualization. Every few seconds the recorded the signal data of each channel is appended. Later, this structure gets transformed into a channel-based representation.

The signal data itself is a sequence of 16 bit integers. However, the data is stored in so-called records, where the measures of all channels in a certain time interval are appended subsequently. The data of a certain channel is thereby spread across a file (Figure 1).

Depending on the number of signals and their sampling rate, file sizes of a whole night PSG in EDF might range from 30 MB to 2 GB, but are usually between 300 and 500 MB^6^.

There is no standard text-based export format for events, such as the sleep stages. EDF+ does allow an “EDF Annotations” signal as a separate channel for storing annotations and events. However, these would be limited in size, binary encoded and bound to the (large) data file, which makes it cumbersome to work with. Therefore, JSON is chosen as the export format for annotation data, where one object contains an unlimited number of events as key-value pairs. The key is a UNIX timestamp^7^ in milliseconds and the value is a string that describes the event.

### 4.2. Signal Compression Technologies

Typically, there is a higher time resolution of the biosignals than the horizontal display resolution. For example, the AASM recommends a sampling rate of 500 Hz for EEG, ECG, EOG, and EMG (Berry et al., 2017), i.e., 500 values within 1 s. In contrast, when displaying an epoch of 30 s on a standard monitor with a horizontal resolution of 1,200 pixels (px), there are 40 px available for the data of each second. Therefore, 12 signal values would be drawn in the same pixel column and overlay during rendering. The same visual result can be obtained by only drawing the minimum and maximum values. Such a compression would lead to a space gain of 80% in the mentioned example while preserving an accurate representation of the signal data (Hadjileontiadis, 2006). The reduced RAM consumption would minimize the risk of crashing the browser tab. However, such compression requires re-parsing of the data if the browser window gets resized or the displayed time range is changed, which may lead to high CPU consumption.

### 4.3. Web Technologies

The solution builds upon modern web technologies. To develop the application in separate components that can be used independently or in composition, React, a JS library for building user interfaces, is utilized. It allows to embed the application or a subset of these components in other applications, build variations of it or extend it with own components. The final code is provided as static files, so no server-side processing is required and any web-server can be used to deploy the application. For biosignal rendering, different drawing methods have been considered, in particular Scalable Vector Graphics (SVG) and the HTML Canvas element. The prevailing opinion is, that Canvas renders faster than SVG (Kee et al., 2012). But this may not be generally the case today and depends on factors like the number of rendered elements (Horak et al., 2018). Based on preliminary performance tests, described in section 4.4.1, a library is chosen that employs the fastest rendering technique, that is then further optimized.

Peer-to-peer (P2P) architectures, where end-users can communicate directly with each other over the internet, as envisioned for the collaboration use case, have been standardized over the last years as Web Real-Time Communication (WebRTC). It is now supported by most browsers (e.g., Chrome, Firefox, Edge, Safari) and allows for audio, video and data communication without any additional plugins. Connections are end-to-end encrypted by default and communication with a server is only necessary for signaling (i.e., initial peer discovery).

### 4.4. Performance Test System

Web applications are difficult to reliably test for performance, especially when they require user interaction, such as selecting a file from the local file system for an input element. And while most browsers include developer tools that help measure application performance, they also require user interaction, for example, to start and stop the profiler and to measure RAM usage or the distribution of computing time over a specific period of time. Both problems have been solved with Puppeteer, a JS library that allows to automatically start, control and profile a Chrome instance. Therefore, a dedicated test system based on Puppeteer is developed to make informed decisions based on real performance data during development of the application. The tests are described as JS scripts, which load test HTML files and precisely measure specific properties like the execution time of certain parts of the code or the overall RAM consumption. All tests are executed on a Mid 2013 MacBook Air with an 1.3 GHz Intel Core i5 and 8 GB of RAM.

#### 4.4.1. Preliminary Performance Tests

To evaluate the performance of different technologies for our particular use case of rendering time series data, three test cases have been implemented: one based on Canvas and two based on SVG, using the Path element and the Polyline element, which are both commonly used to render graphs. Each test case is additionally split into two versions, one for single uncompressed values and one for min-max pairs. The test data consists of 1,200 random data points per channel, generated in advance. To further mitigate any unknown browser-internal optimizations, two different sets of the data have been generated and each draw uses a random mix of both sets.

A common problem in testing rendering performance is to only measure JavaScript execution time, thus missing the time the browser takes to calculate styles, lay out the changes and paint them, which also blocks the main thread. Therefore, each draw is queued via setTimeout to let the browser finish all work before continuing. Each implementation was tested for 8, 16, 32, and 64 channels with 100 draws each. For every test, a trace was recorded and evaluated for the time spent on scripting, painting and rendering.

### 4.5. Integration Into Data Analytics Environment

XNAT^8^ is a free and open-source web-based data management system developed to support clinical trials and offers structured storage capabilities for popular formats in neuroscience (Marcus et al., 2007). It can be easily extended to support other formats like EDF (Beier et al., 2017). Data is mainly organized in a hierarchy of projects, subjects, and experiments. XNAT provides a user management system with fine-grained access control capabilities for the stored data. Users authenticate with username and password and receive an authorized token within a cookie that is valid for the current session. XNAT offers a comprehensive REST API, which makes it easy for other applications to interact with it over the web. XNAT also includes a so-called pipeline engine that allows for arbitrary data analysis by defining an analysis pipeline, a sequence of subsequent tasks that are stored in XML-based pipeline definition. It is possible to call executables within these pipelines that can receive parameters. These can be passed in three different ways: dynamically (based on the current context), automatically (as pre-defined values) and manually (via the user interface or REST call).

In our context, XNAT is used to store EDF files, analysis results, and related artifacts. The presented application can upload data to XNAT and visualize automatically detected events. To allow communication between both, the same-origin policy (SOP) has to be respected. The SOP is a fundamental security model of the web, that restricts JS scripts to only access data served under the same origin, i.e., the combination of protocol, host, and port. Therefore, the application is integrated into the Apache Tomcat instance that also runs XNAT. All communication between them is encrypted.

### 4.6. Open Development

The application is developed under the MIT license. All source code is hosted on GitHub under github.com/somnonetz/copla-editor which can be freely used and extended. The repository also includes all test files and sample results. This makes it easy to inspect, reproduce and compare the results. It is therefore also possible to deploy and use the application in systems without access to the internet, e.g., a hospital intranet.

## 5. Implementation

The application's core consists of two distinct parts: a parser and a visualizer. A collaboration add-on utilizes this core to enable live peer-to-peer communication with another user.

To be able to also use the application offline, it is fully loaded and cached the first time it is accessed by the user. This is possible by leveraging a Service Worker, a script that acts as an intermediate between other scripts and the browser, and can handle network requests and fulfill them with data from a cache in case no network is available.

From then on, a network connection is only needed to view remote files or to collaborate. EDF files can be opened by providing a URL to a remote file via a query parameter or by drag and drop of local files. Channel names are automatically substituted with their equivalent from the EDF+ standard to provide a homogeneous view to the user.

To achieve a fast FMP, the application first loads only the headers and the signal data for the application's current window size, the so-called viewport. Then a buffer of two viewport widths left and right of the currently shown signal part is preloaded and cached. From then on the cache constantly provisions new data for time ranges that are likely to be viewed next and removes data that has become dispensable, when a user moves through the file.

### 5.1. Parser

The parser converts EDF data into a JS data structure. Data values in EDF have implicit time information based on their position in the file. To make it easier to address time points within the data, the parser converts each value into a tuple containing the value and its absolute time as a JS Date object. Due to its high RAM consumption, this solution is not feasible to parse a whole PSG of 300 MB in the browser. But since EDF arranges data chronologically, it is possible to only load a specific segment of a file to provide parsed data for a certain time range. Therefore, the parser has been build to be able to parse EDF segments of arbitrary length (rounded to the nearest record).

The parser itself offers the asynchronous method getData, that receives a time range and optionally a frequency parameter. The frequency describes the maximum number of values the resulting data should contain per second. The parser then compresses the signal data by reducing all values of a certain time range to its minimum and maximum values. The time range parameter is internally translated into its corresponding byte range, i.e., the exact position of the first and last byte in the file.

To decouple the parser from a concrete data source, it depends on an abstract loading strategy. This can be any JS object that implements a read method. This method receives a byte range from getData and returns a Promise object that will resolve with the corresponding EDF data. Three loading strategies have been implemented, for local files, remote files and a WebRTC peer. To support partial loading, the local strategy relies on the ability of the FileReader API to read slices of a file. The remote strategy in contrast uses HTTP range requests that must be supported by the web server that hosts the file.

### 5.2. Visualizer

**Figure 2:**
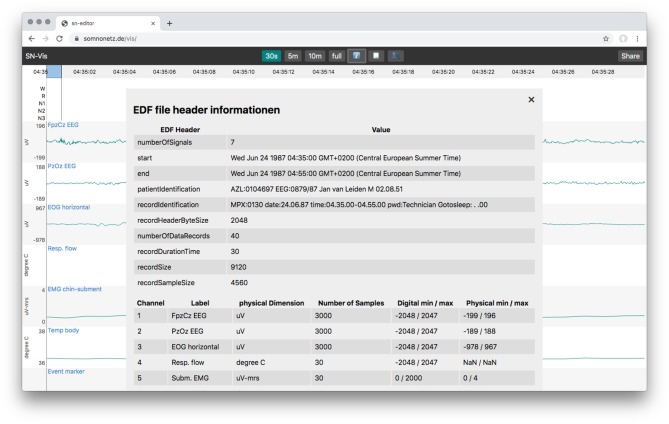
Overlay with the EDF file header information, showing the static and the dynamic header data.

The user interface mimics those of other applications used in sleep labs. It displays the signal graphs of all channels, supports scoring, has controls to switch between common time windows (e.g., 30 s, 5 min, full) and allows to inspect a files header information (Figure 2).

#### 5.2.1. Preliminary Performance Test Results

**Figure 3:**
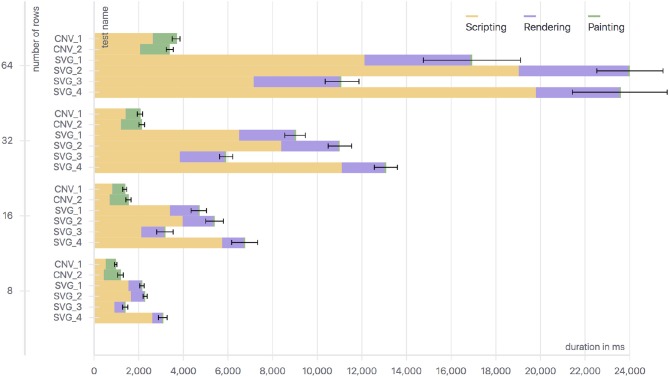
Runtime data for different SVG and Canvas implementations drawing different amounts of signals (rows) using single values and min-max pairs as data. *CNV_1*, Canvas with min-max-pairs; *CNV_2*, Canvas with single values; *SVG_1*, SVG using multiple path elements and min-max pairs; *SVG_2*, SVG using one path element for all min-max pairs; *SVG_3*, SVG using multiple path elements and single values; *SVG_4*, SVG using a polyline element and single values.

The results of the tests, as described in section 4.4.1, are presented in Figure 3. It shows the overall time needed for several implementations using different approaches (Canvas and SVG) and data structures (single values and min-max pairs) to draw the given test data for 8–64 signals (rows) 100 times each. The drawing time is divided into the different categories of browser work: scripting, rendering and painting. Scripting refers to the computational time spent on executing script code. Rendering includes computation of styles associated with HTML elements and their positioning in the layout. Painting refers to the time needed to rasterize graphical data and draw it. The Canvas based implementations spent no time on rendering, as the same HTML element is re-used. The SVG versions on the other hand spent nearly no time on painting because previous data points can be reused, but they need to be re-positioned, which is why more time is needed for the rendering step.

The most relevant tests regarding our use case are *CNV_1* (Canvas with min-max-pairs) and *SVG_2* (SVG using min-max-pairs). The Canvas implementation performed best in every configuration. It is about three times faster for 16 channels and about five times faster for 64 channels.

Based on these results, the open source JS library dygraphs^9^ was chosen, as it uses a Canvas element to plot data. It supports panning and zooming, handles user input (e.g., dragging elements with the mouse), automatically enriches the chart axes with value labels, and offers utility functions (e.g., to translate pixel coordinates to timestamps). It also offers are plugin API. However, dygraphs showed some performance issues when updating a graph frequently, therefore the library was forked and optimized for our particular use case. The changes include (ordered according to the impact on the rendering performance):

- Reusing HTML elements by introducing an element pool to save time from frequent destruction and recreation, e.g., for timestamp labels)
- Memorizing values calculated in loops, deterministic function call results, and colors (color strings like "red" were translated into RGB by applying them to a hidden HTML element and then reading the resulting color code from it)
- Using binary search instead of linear search to find the indices of the first and last data points of the currently visible viewport
- Replacing CPU based positioning of timestamps by a GPU-based method
- Not updating HTML elements that are hidden or whose content didn't change, such as the legend or graph labels)

Furthermore, a dygraphs plugin has been developed to support the visualization and handling of events within the biosignals [requirement (d)]. Events can be added manually by selecting a time range in a channel and then add a label. The plugin supports shortcuts for common apnoe labels^10^. Events can also be resized, moved, and removed. Existing events can be imported and are immediately added to the visualization.

### 5.3. Collaboration Add-on

**Figure 4:**
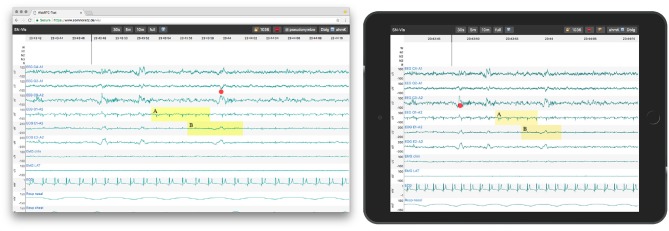
A PSG session in Google Chrome on MacOS (left, host) and an emulated tablet (right, peer). Both parties see the same data range and the marked events. The mouse position of the other party is shown as a red dot.

The application enables collaboration with one other party utilizing WebRTC. To start a collaboration session, one party has to load a local or remote EDF file, as described before. A share button appears, which, when clicked, generates a PIN and shows a URL containing it. These have to be shared with the other party over a second medium, e.g., phone or email. The other party then opens the URL or manually enters the PIN to join. Thereafter, all actions are synchronized and both parties act with the same priority. To ease the communication, each user also sees the live position of the mouse pointer of its counterpart as a red dot (see Figure 4). Both users can also initiate a call directly in the application (via a click on the phone icon in the header), to establish an audio channel.

There is not functionality to resolve conflicting behavior yet, e.g., if both parties move in different directions at the same time. Such conflicts currently have to be solved on the social level, e.g., by communicating ones intent via the audio channel.

**Figure 5:**
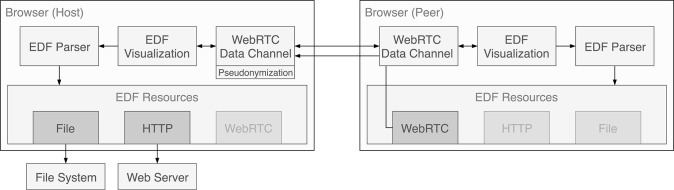
Message and data flow within the applications and between the two instances in the collaboration setting. Data sources are always handled through the parser. To enable visualization of data provided by a peer, the cached data is sent over the WebRTC data channel and is presented to the parser of the receiving instance as a WebRTC data source.

Technically, the sharing behavior is implemented as another loading strategy of the parser (see Figure 5). When a client requests data from the resource, it is transparently transferred from a connected peer.

As in the case of the other loading strategies, still only the currently needed segments of the EDF file are transferred. The app sends raw EDF data instead of serializing and sending parsed data as it turned out to be much faster and mitigates compression problems for diverging display resolutions. The data is not persisted by default. However, to allow file sharing the receiving party has a button to download the entire file.

### 5.4. Pseudonymization

To account for patient data protection, the application pseudonymizes each file locally before network transfer by default. Pseudonyms are randomly generated strings. The application also handles their management, import and export: All pseudonyms are put in a persistent key-value store in the browser, the so-called Local Storage. The mapping of patient data and pseudonyms can be exported as an Excel file^11^. They can also be imported in the same format, e.g., to re-pseudonymize remotely stored EDF files. The default behavior can be disabled by the sending party giving the user the full control over the privacy level.

### 5.5. Integration With Data Management

**Figure 6:**
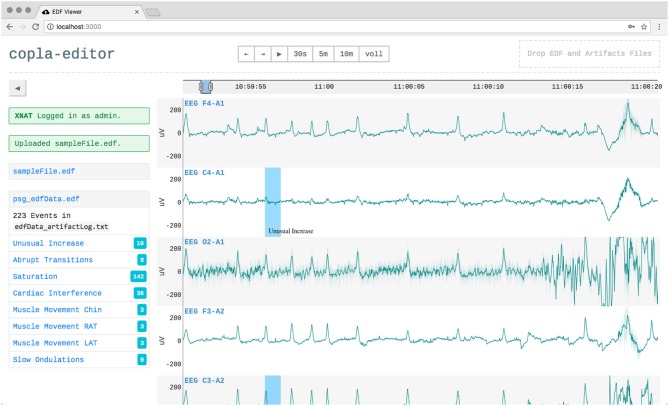
The user interface when a user is logged in to XNAT and had uploaded a file, which was automatically analyzed. Detected events like artifacts (blue areas) were played back into the visualization.

The application allows users to log in to a remote XNAT instance and upload local EDF files. The files are then automatically analyzed in the platform as a feature of XNAT. These analyses can produce events, e.g., detected artifacts or apnea episodes, which are then pulled back by the application and are used to enrich the local visualization (see Figure 6) (Witt et al., 2017).

## 6. Performance Optimization

The visualization library dygraphs provides many needed features out of the box, but its original use case is different from ours and some of its constraints turned out to be suboptimal for the proposed application regarding RAM usage and rendering performance. The application has achieved an FMP of about 1100 ms. This includes loading the site with empty cache from a remote server (440 ms), loading a remote EDF file (450 ms) and rendering the first epoch (210 ms). It updates with about 7 frames per second in constant scrolling, resulting in L = 143 ms. This is acceptable but does not reach the envisioned update time of below 100 ms, and leads to notable lagging if one scrolls quickly through the data. Therefore, alternative approaches have been tested that show better performance. Although they were finally not employed, because they either were not compatible with dygraphs or did not meet requirement (f) as they depend on modern browser features, the attempts are here described for completeness and as starting point for further improvements. All implementations and tests and available in the application's GitHub repository.

### 6.1. RAM Consumption

Dygraphs requires time series data to be specifically structured^12^. Each entry has to be an array containing a JS Date object, the value and in our case, when compression is used, an additional array containing the min and max values. Unfortunately, parsing EDF into a JS representation with this structure leads to a substantial increase in space usage: Parsing 10 MB of EDF results in 1 GB of RAM usage and takes about 5 s. Replacing the Date objects with timestamps as numbers reduces the RAM usage to 440 MB and parsing time to 890 ms. A full PSG of 300 MB would therefore theoretically need 52 s to parse while consuming 13 GB of RAM instead of 150 s and 30 GB of RAM without optimization. Compression mitigates this problem and parsing 10 MB EDF data with a frequency of 40 decreases RAM usage to 168 MB with a similar parsing time of 780 ms. However, RAM consumption and parsing times could be even further reduced to 430 ms and 61 MB (for 10 MB with a frequency of 40) by not using a separate array for each entry, but one flat array for all data of a channel with a repeating sequence of the three numbers: timestamp, min value and max value. This representation would be feasible to hold many more data without risking to crash the browser tab, but it would require a substantial modification of the visualization library.

### 6.2. Rendering Performance

**Figure 7:**
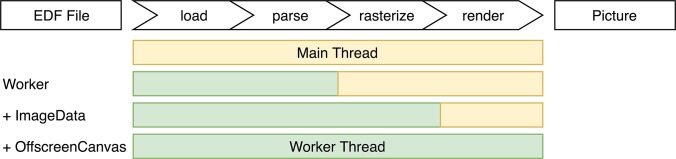
All distinct steps involved from raw data to final picture, and different optimization approaches investigated.

One way to increase rendering performance is to offload as much work as possible to a Web Worker, a thread like model that allows for concurrent code execution (Verdu and Pajuelo, 2016). They follow the actor model, so they don't share state and communicate asynchronously over messages. All steps in Figure 7 are candidates for offloading to a Web Worker. As parsing and rendering are the most computational expensive parts, they were targeted first. Unfortunately the dygraphs library is tightly coupled with the HTML element structure, which is not available in a Web Worker thread.

If only the parser runs in a Web Worker, the overhead for transferring the result becomes more expensive than the parsing itself, because Web Workers use structured cloning to transfer complex data. Loading 10 MB EDF data (frequency of 40; timestamps as Date objects) from a Web Worker took 2,340 ms with 1,080 ms spent on the transfer.

To mitigate this kind of problem, Shared Array Buffer (SAB) was introduced to the web platform. A SAB is a fixed-length raw binary data buffer. It is accessible to both the Web Worker and the main thread without cloning. However, they do not support complex data types, so it would have been necessary to fully flatten the compressed EDF data and adjust the visualization library accordingly. Beyond that, SABs were disabled in Chrome at the time of development (and re-enabled since), Firefox (now behind a feature toggle which is turned off by default and needs to be explicitly enabled by the user) and Edge to mitigate the speculative execution side-channel attacks Meltdown and specter.

To make it easier to transfer data between a Web Worker and the main thread, browsers also introduced Transferable Objects, which are not prone to structured cloning and can be passed much faster. One of these objects is Image Data, a representation of pixel data for a Canvas element. They can be used to handle loading, parsing and rasterization in a Web Worker and transfer the results back to the main thread with nearly no overhead (around 3.7 ms for an 1,200 × 800 px image).

Lastly, the currently experimental Offscreen Canvas was evaluated, which provides a Canvas that can be rendered off screen, especially in a Worker. It showed the best performance with nearly no overhead for transfer. However, it is currently only supported in Chrome and Firefox (behind a feature toggle).

A different optimization approach investigated is tiling. Instead of re-rendering all graphs while scrolling, one dedicated Canvas, i.e., a tile, is rendered for each time range. These tiles are placed next to each other in a row and the browsers normal scroll behavior is used for movement. To manage the number of tiles, the library “react-window” was used to render the whole EDF time span as a virtual list of tiles each as wide as one viewport width. It renders up to four tiles in advance and uses Offscreen Canvases to offload work. The application runs at the desired 60 frames per second, with nearly no workload on the main thread, and stays responsive to user input.

Again, dygraph does not support this behavior. In case an own visualization solution would be developed to replace dygraphs, it should use tiling. Beyond that, especially Image Data and Offscreen Canvas are promising potential optimizations.

The code for all mentioned optimization efforts can be found in the “performance-tests” directory in the GitHub repository.

## 7. User Acceptance Testing

To evaluate whether the proposed solution meets the user requirements, user acceptance tests have been conducted with partners from different backgrounds (medical technical assistants, clinicians, and sleep researchers) and different facilities in multiple cities in Germany.

The test scenario consisted of several steps to determine if:

1. a user can open a local EDF file
2. a user can open an EDF file from XNAT when logged in
3. a user can inspect an opened file
4. a user can move within the timespan of a file
5. a user can mark events and manipulate them
6. a user can download the annotations
7. a user can share a file and gather an PIN
8. a user can connect with a host
9. a user can call the other user
10. data shared over a connection is pseudonymized
11. data is synchronized between connected users, especially the EDF data, the time resolution, the viewport, event data and the mouse cursor
12. a user can download the shared EDF file
13. a host can download all pseudonyms along with the original patient data

The tests were carried out with five participants. Initially, all participants were called via phone and instructions were given orally. Once step 9 was reached, the phone call was ended and both parties switched to the app internal audio channel. All participants were able to successfully complete all test steps. In one case, a network error occurred, presumably due to proxy settings of the hospital network; this was mitigated by tethering over a mobile hotpot and the test could then be continued. All tests were carried out successfully and the participants were able to complete the given tasks after a short introduction. All participants answered yes to the questions as to whether in their opinion the application was intuitive to use and whether they were satisfied with the performance. While the local usage has shown to be robust within different hardware, operating systems and browsers, collaboration depends on a stable and non-restrictive internet connection.

## 8. Conclusion and Outlook

The presented application enables clinicians and researchers to assess biosignals in EDF and to collaborate with others, while protecting sensitive patient data. Implemented as a web application, it runs without installation and on all modern browsers. The application was presented at the annual congresses of both the GMDS (German Association for Medical Informatics) and the DGSM (German Sleep Society). The DGSM plans to freely host the application for its members to foster communication between researchers, clinicians and patients. The most requested feature was video support (patients are usually filmed during sleep to ease signal interpretation), in particular for teleconsultation, to discuss the data and the resulting diagnoses with patients, especially in rural or less urban areas. However, video poses data protection issues, as patient's faces are typically seen in the videos. Furthermore, higher data transfer rates are required to reach the accepted performance, and synchronicity between biosignal and video data is difficult to reach, as typically recorded with different non-synchronized devices.

The application is also envisioned to be used it in a web-based learning platform to train medical-technical assistants for functional diagnostics by scoring against expert's assessments. This would not only make the trainees more independent about the time, place and duration of their training; but would also help, as the need for close proximity between trainer and apprentice is expensive and inhibiting. Support for more than two peers is possible and envisioned, but full synchronization between many peers might lead to lags and the number of editing users may need to be restricted to ensure the stability of the tool. Regarding online repositories like sleepdata.org and physionet.org that offer free access to biosignal recordings, the application could be used to allow inspection of recordings without the need to download them. The integration of analysis methods, such as filters, frequency analysis or automatic artifact detection would further enhance the usability of the application.

## Data Availability Statement

The datasets generated for this study can be found in the GitHub repository at github.com/somnonetz/copla-editor.

## Author Contributions

MB developed the application and ran the experiments. DK and MB wrote the article. TP reviewed the article.

### Conflict of Interest

The authors declare that the research was conducted in the absence of any commercial or financial relationships that could be construed as a potential conflict of interest.

## Footnotes

^1^Depending on the variation; 3 × EEG, 2 EOG, 2 × chin EMG, 2 × leg EMG, 2 × airflow (nose, mouth), 2 × respiratory effort, blood oxygen saturation, body position, ECG.

^2^http://www.edfplus.info

^3^e.g., LightWAVE: https://physionet.org/lightwave, Altamira: https://github.com/nsrr/altamira, Interactive EEG Atlas: http://www.atlas.ansuk.org

^4^https://github.com/BilalZonjy/EDFViewer

^5^https://github.com/jusjusjus/edfjs

^6^Based on data from https://sleepdata.org/datasets (Zhang et al., 2018).

^7^A way to describe a point in time by the number of seconds that have elapsed since the 1st of January 1970.

^8^https://www.xnat.org

^9^https://github.com/danvk/dygraphs

^10^H, hypopnoe; Z, central apnoe; G, mixed apnea; O, obstructive apnea.

^11^Excel may not seem like a good technical choice, but in our experience it is the format most clinicians can work with efficiently.

^12^http://dygraphs.com/data.html#array

## References

[B1] Anderson R. J. (1996). Security in Clinical Information Systems. London: British Medical Association.

[B2] Andrikos C. Rassias G. Tsanakas P. Maglogiannis I. (2019). An enhanced device-transparent real-time teleconsultation environment for radiologists. IEEE J. Biomed. Health Inform. 23, 374–386. 10.1109/JBHI.2018.2824312 29993993

[B3] Andriopoulou F. G. Birkos K. Lymberopoulos D. (2015). P2care: a dynamic peer-to-peer network for collaboration in personalized healthcare service delivery. Comput. Ind. 69, 45–60. 10.1016/j.compind.2014.09.007

[B4] Beier M. Jansen C. Mayer G. Penzel T. Rodenbeck A. Siewert R. (2017). Multicenter data sharing for collaboration in sleep medicine. Fut. Gen. Comput. Syst. 67, 466-480. 10.1016/j.future.2016.03.025

[B5] Berry R. B. Brooks R. Gamaldo C. Harding S. M. Lloyd R. M. Quan S. F.. (2017). AASM scoring manual updates for 2017 (version 2.4). J. Clin. Sleep Med. 13, 665–666. 10.5664/jcsm.6576 28416048 PMC5406946

[B6] Combrisson E. Vallat R. Eichenlaub J.-B. O'Reilly C. Lajnef T. Guillot A.. (2017). Sleep: an open-source python software for visualization, analysis, and staging of sleep data. Front. Neuroinform. 11:60. 10.3389/fninf.2017.00060 28983246 PMC5613192

[B7] Goldberger A. L. Amaral L. A. N. Glass L. Hausdorff J. M. Ivanov P. C. Mark R. G. (2000). PhysioBank, PhysioToolkit, and PhysioNet: components of a new research resource for complex physiologic signals. Circulation 10, E215–E220. 10.1161/01.cir.101.23.e215 10851218

[B8] Hadjileontiadis L. J. (2006). Biosignals and compression standards, in M-Health, eds Istepanian R. S. H. Laxminarayan S. Pattichis C. S. (Boston, MA: Springer US), 277–292.

[B9] Horak T. Kister U. Dachselt R. (2018). Comparing Interactive Web-Based Visualization Rendering Techniques. TU Dresden: Interactive Media Lab.

[B10] Ibáñez V. Silva J. Cauli O. (2018). A survey on sleep assessment methods. PeerJ 6:e4849. 10.7717/peerj.4849 29844990 PMC5971842

[B11] Iber C. Ancoli-Israel S. Chesson A. Quan S. F. (2007). The AASM Manual for the Scoring of Sleep and Associated Events: Rules, Terminology and Technical Specifications. Westchester, IL: American Academy of Sleep Medicine.

[B12] Kee D. E. Salowitz L. Chang R. (2012). Comparing Interactive Web-Based Visualization Rendering Techniques. Medford, MA: Tufts University.

[B13] Kemp B. Olivan J. (2003). European data format ‘plus' (EDF+), an EDF alike standard format for the exchange of physiological data. Clin. Neurophysiol. 114, 1755–1761. 10.1016/S1388-2457(03)00123-8 12948806

[B14] Kemp B. Roessen M. (2007). Polyman: a free(ing) viewer for standard edf(+) recordings and scorings, in Sleep-Wake Research in the Netherlands, eds Ruijgt G. S. F. De Boer T. Van Kasteel V. van Luijtelaar G. Overeem S. (Amsterdam: Dutch Society for Sleep-Wake Research), 71–73.

[B15] Kemp B. Värri A. Penzel T. Olivan J. (2003). Standard Texts and Polarity Rules.

[B16] Kemp B. Värri A. Rosa A. C. Nielsen K. D. Gade J. (1992). A simple format for exchange of digitized polygraphic recordings. Electroencephalogr. Clin. Neurophysiol. 82, 391–393. 10.1016/0013-4694(92)90009-7 1374708

[B17] Körner M. (2010). Interprofessional teamwork in medical rehabilitation: a comparison of multidisciplinary and interdisciplinary team approach. Clin. Rehabil. 24, 745–755. 10.1177/0269215510367538 20530646

[B18] Krefting D. Canisius S. Hoheisel A. Loose H. Tolxdorff T. Penzel T. (2013). Grid based sleep research–analysis of polysomnographies using a grid infrastructure. Fut. Gen. Comput. Syst. 29, 1671–1679. 10.1016/j.future.2010.03.008

[B19] Maglogiannis I. Delakouridis C. Kazatzopoulos L. (2006). Enabling collaborative medical diagnosis over the internet via peer-to-peer distribution of electronic health records. J. Med. Syst. 30, 107–116. 10.1007/s10916-005-7984-1 16705995

[B20] Marcus D. Olsen T. R. Ramaratnam M. Buckner R. L. (2007). The extensible neuroimaging archive toolkit–an informatics platform for managing, exploring, and sharing neuroimaging data. Neuroinformatics 5, 11–34. 10.1385/NI:5:1:11 17426351

[B21] Naït-Ali A. (Ed.). (2009). Advanced Biosignal Processing. Berlin; Heidelberg: Springer Berlin Heidelberg.

[B22] Puel A. Wangenheim V. A. Meurer M. I. Macedo D. D. D. J. (2014). Bucomax: collaborative multimedia platform for real time manipulation and visualization of bucomaxillofacial diagnostic images, in IEEE 27th International Symposium on Computer-Based Medical Systems (New York, NY).

[B23] Roebuck A. Monasterio V. Gederi E. Osipov M. Behar J. Malhotra A.. (2014). A review of signals used in sleep analysis. Physiol. Meas. 35, R1–R57. 10.1088/0967-3334/35/1/R1 24346125 PMC4024062

[B24] Semmlow J. (2012). The big picture, in Signals and Systems for Bioengineers, ed Bronzino J. (Hartford, CT: Elsevier; Trinity College), 3–33.

[B25] Sherif T. Rioux P. Rousseau M.-E. Kassis N. Beck N. Adalat R.. (2014). CBRAIN: a web-based, distributed computing platform for collaborative neuroimaging research. Front. Neuroinform. 8:54. 10.3389/fninf.2014.00054 24904400 PMC4033081

[B26] Verdu J. Pajuelo A. (2016). Performance scalability analysis of JavaScript applications with web workers. IEEE Comput. Archit. Lett. 15, 105–108. 10.1109/LCA.2015.2494585

[B27] Witt M. Jansen C. Breuer S. Beier M. Krefting D. (2017). Artefakterkennung über eine cloud-basierte Plattform. Somnologie 21:2067 10.1007/s11818-017-0138-0

[B28] Zhang G.-Q. Cui L. Mueller R. Tao S. Kim M. Rueschman M.. (2018). The National Sleep Research Resource: towards a sleep data commons. J. Am. Med. Inform. Assoc. 25, 1351–1358. 10.1093/jamia/ocy064 29860441 PMC6188513

